# Supplementary figures and images for: Bivalent individualization during chromosome territory formation in Drosophila spermatocytes by controlled condensin II protein activity and additional force generators
Source: PLoS Genet. 2021 Oct 20;17(10):e1009870. doi: 10.1371/journal.pgen.1009870 (PMC8559962; doi:10.1371/journal.pgen.1009870)

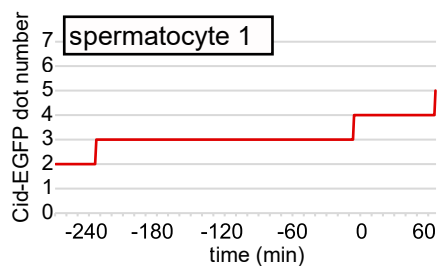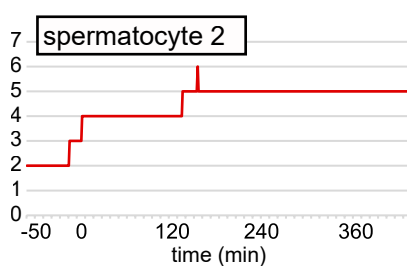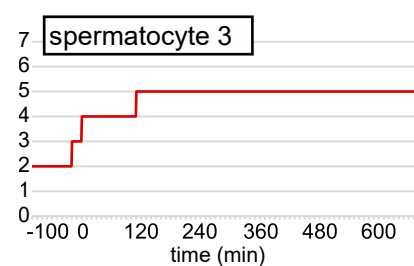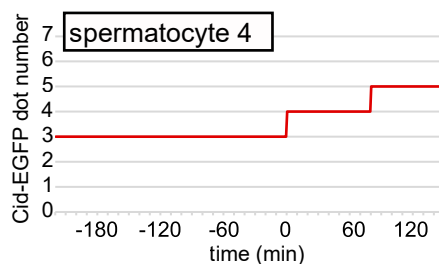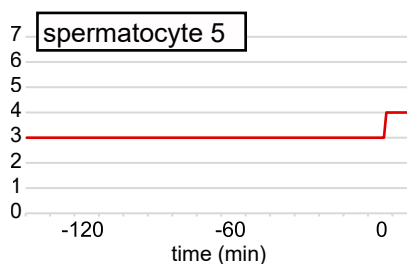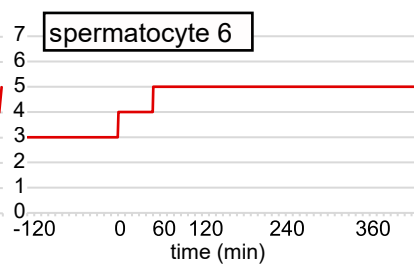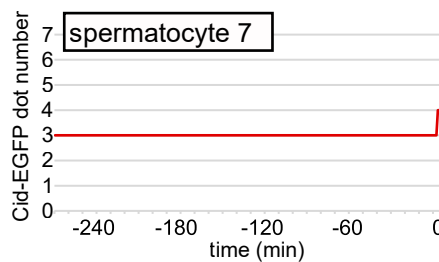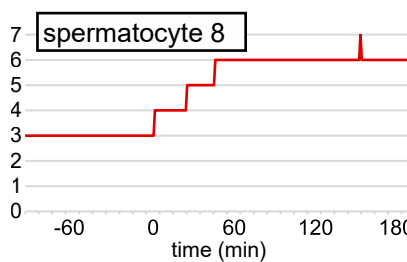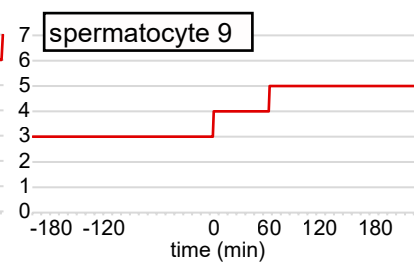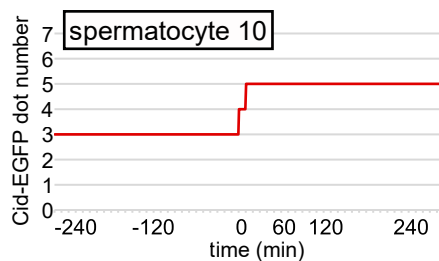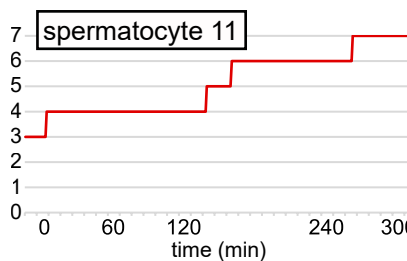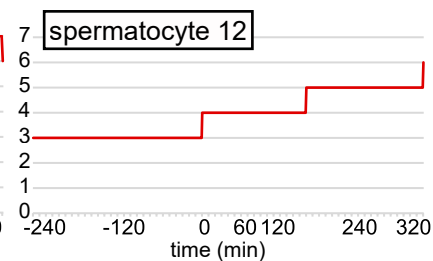

Supplement: S1 Fig — Spermatocytes expressing His2Av-mRFP and Cenp-A/Cid-EGFP were analyzed by time-lapse imaging. The number of centromeric Cid-EGFP dots was determined and plotted over time. The graphs from a subset of the analyzed spermatocytes are displayed for illustration of the considerable temporal variability of centromere de-clustering. t = 0 was set at the transition from the three- to the four-dot stage. (PDF) [file pgen.1009870.s001.pdf]

**A**

S1/2

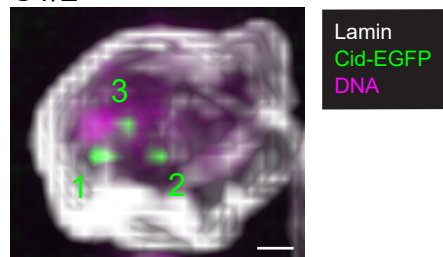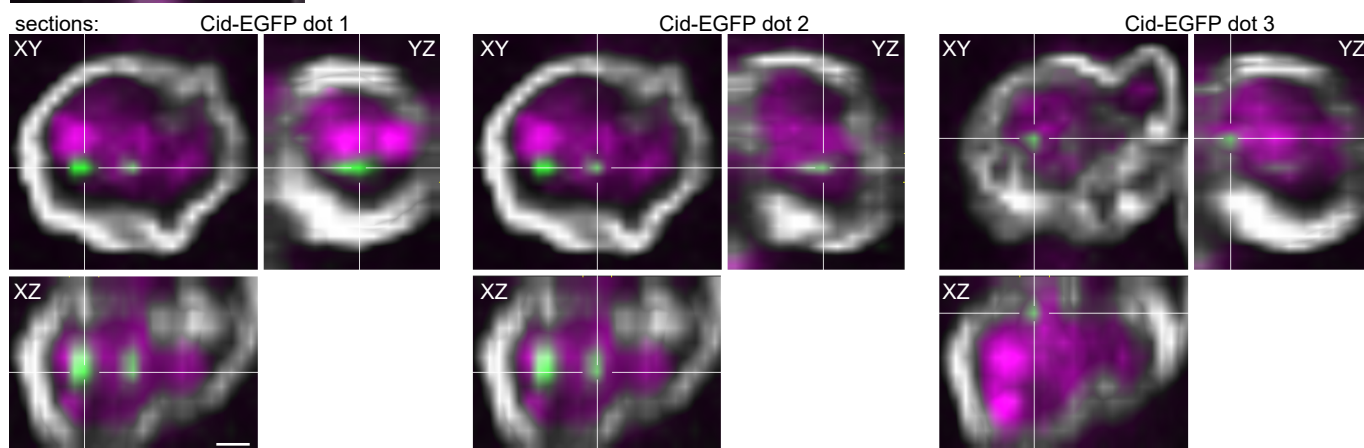

S3

S4

S5

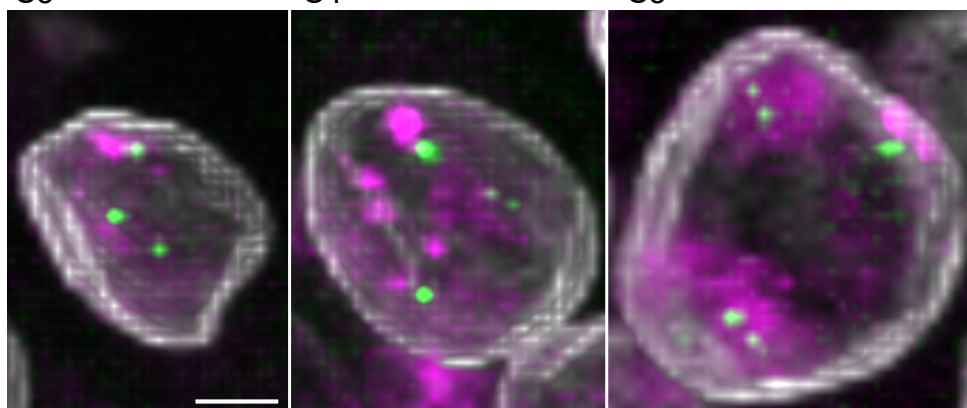**B**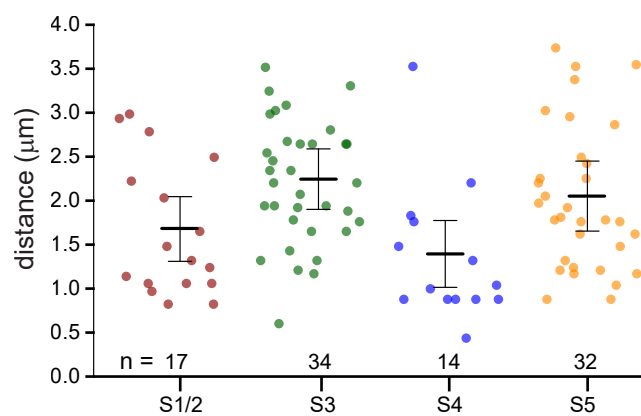

Supplement: S2 Fig — (A) Whole mount testis preparations were labeled with anti-Lamin Dm0 and a DNA stain. Maximum intensity projections with representative spermatocytes at the indicated stages illustrate that the large majority of Cid-EGFP dots is not intimately associated with the nuclear lamina. In case of the S1/2 spermatocyte with three Cid-EGFP dots (top), optical sections cutting through these dots are presented below the projection. (B) Dot plot presenting the separation distance between Cid-EGFP dots and the nuclear lamina at the indicated stages. The number of the analyzed dots is given in the plot, as well as the mean distance (± s.d.). Number of spermatocytes analyzed = 6 (S1/2), 9 (S3), 4 (S4), and 5 (S5). Scale bars = 1 μm (S1/2) and 3 μm (S3 –S5). (PDF) [file pgen.1009870.s002.pdf]

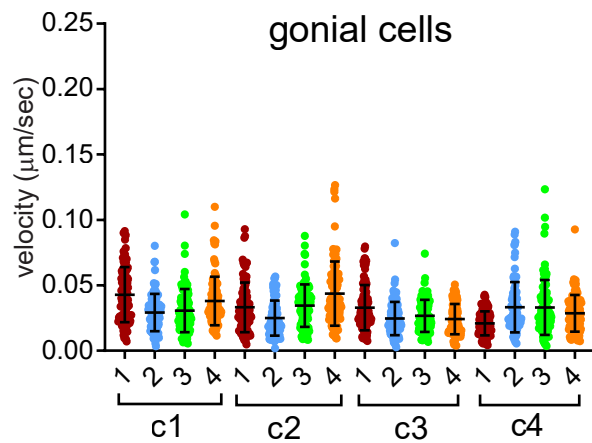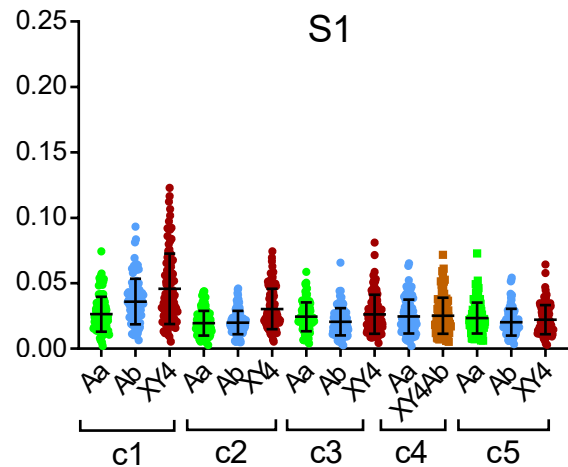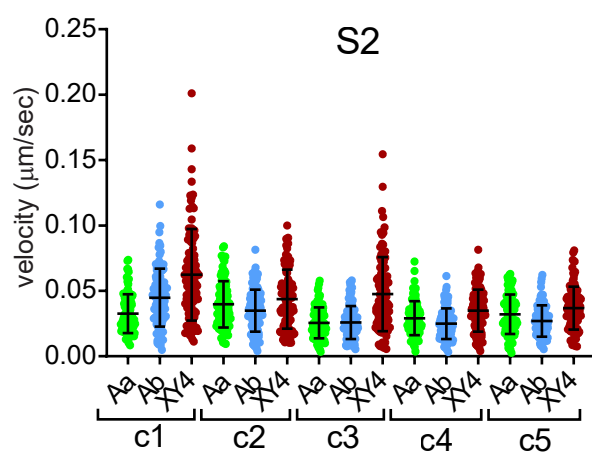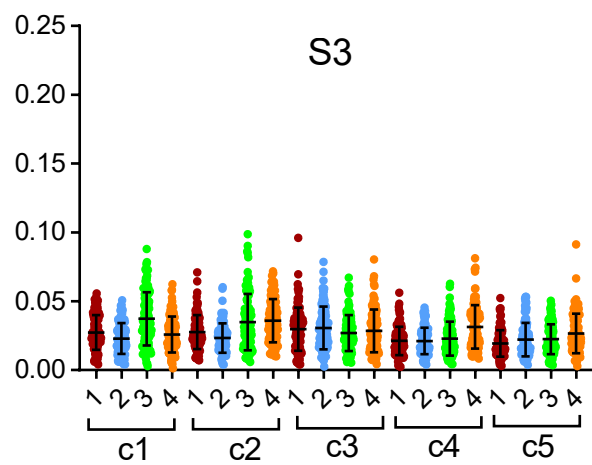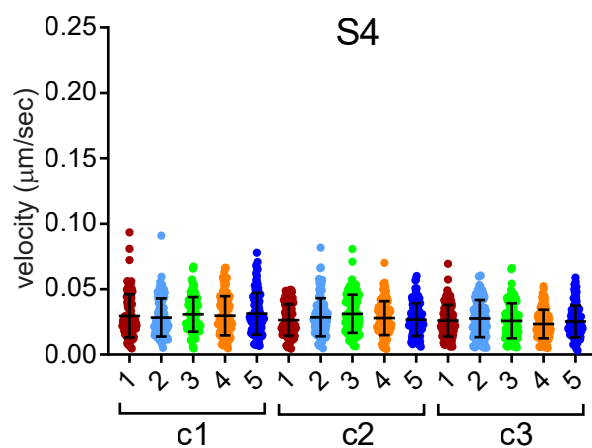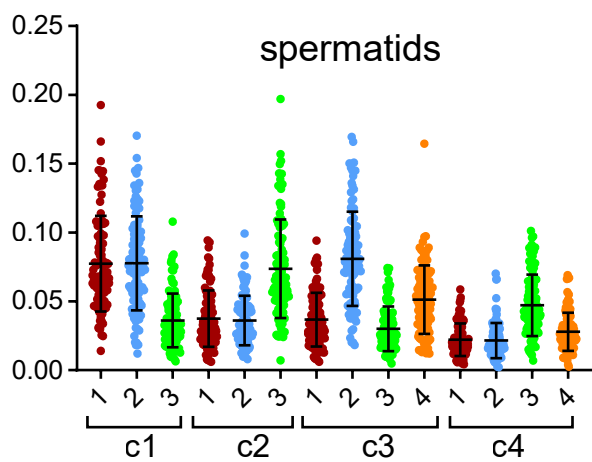

Supplement: S3 Fig — Testes expressing Cenp-A/Cid-EGFP and His2Av-mRFP were used for time-lapse imaging at five-seconds intervals. Three to five cells (c1-c5) at the indicated stages were analyzed. Each Cid-EGFP dot in these cells was tracked over time. Dots were numbered, except for the stages S1 and S2, where designations as in Fig 1A were applied. All velocity values observed for a given Cid-EGFP dot during an eight min period were plotted (n = 99), as well as the mean ± s.d. (PDF) [file pgen.1009870.s003.pdf]

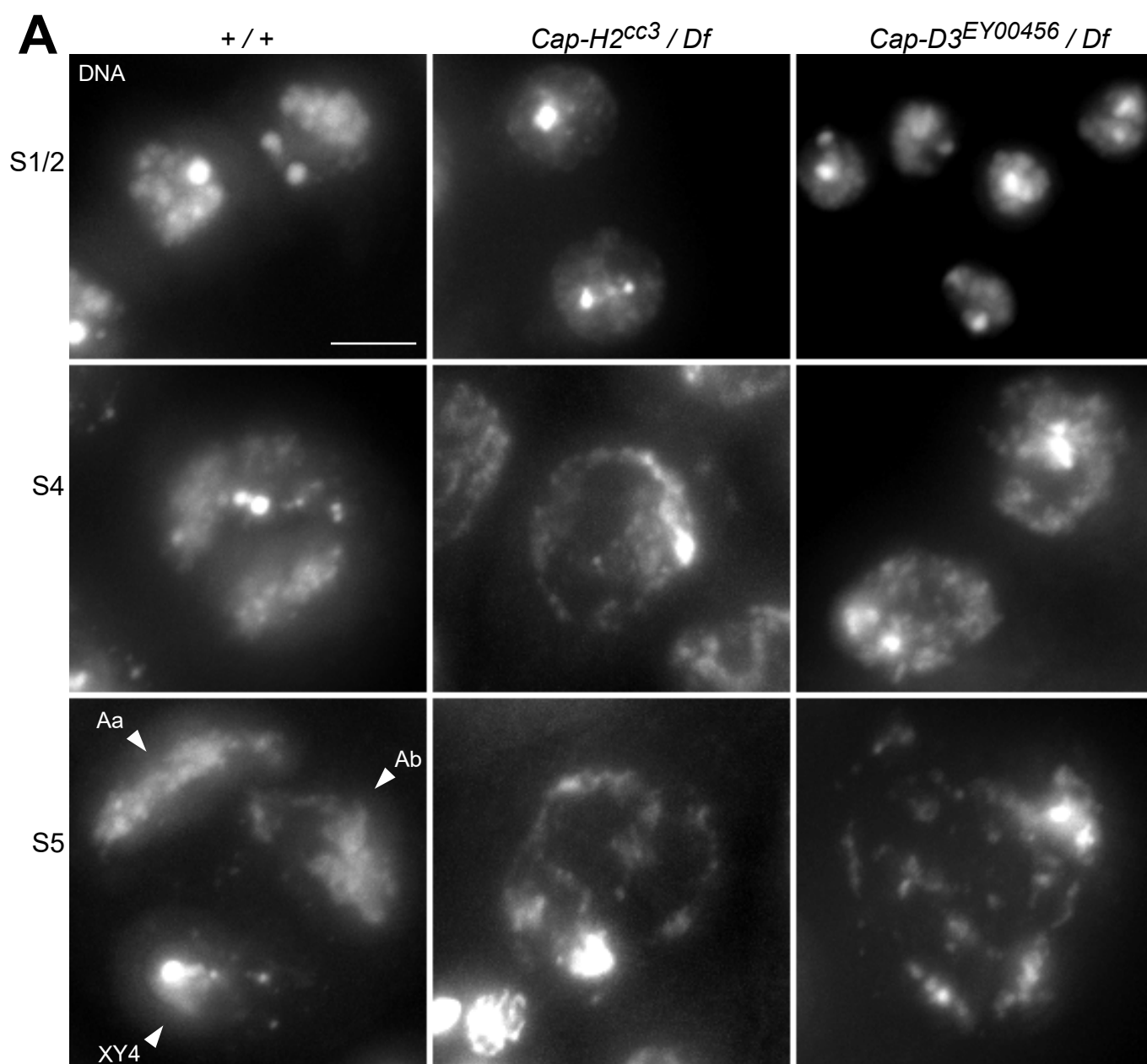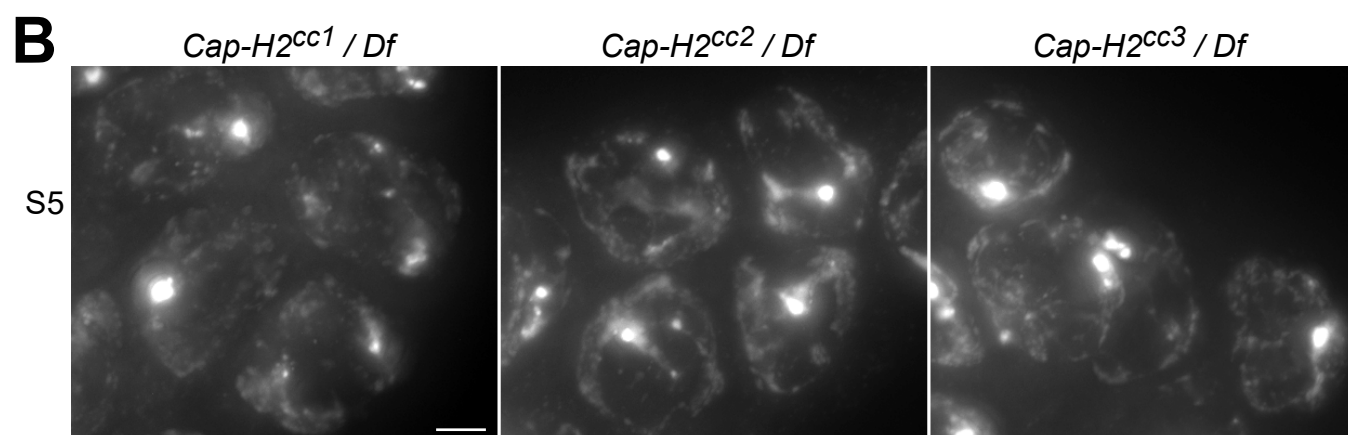

Supplement: S4 Fig — (A,B) Squash preparations of testes with the indicated genotypes were stained for DNA. Spermatocytes at the indicated stages are displayed. In control spermatocytes (+ / +), the three chromosome territories containing either large autosomes (Aa and Ab) or the other chromosomes (XY4) are evident at the S4 and even more clearly at the S5 stage (arrowheads). In contrast, territories are absent in Cap-H2 and Cap-D3 mutant spermatocytes. Scale bars = 5 μm. (PDF) [file pgen.1009870.s004.pdf]

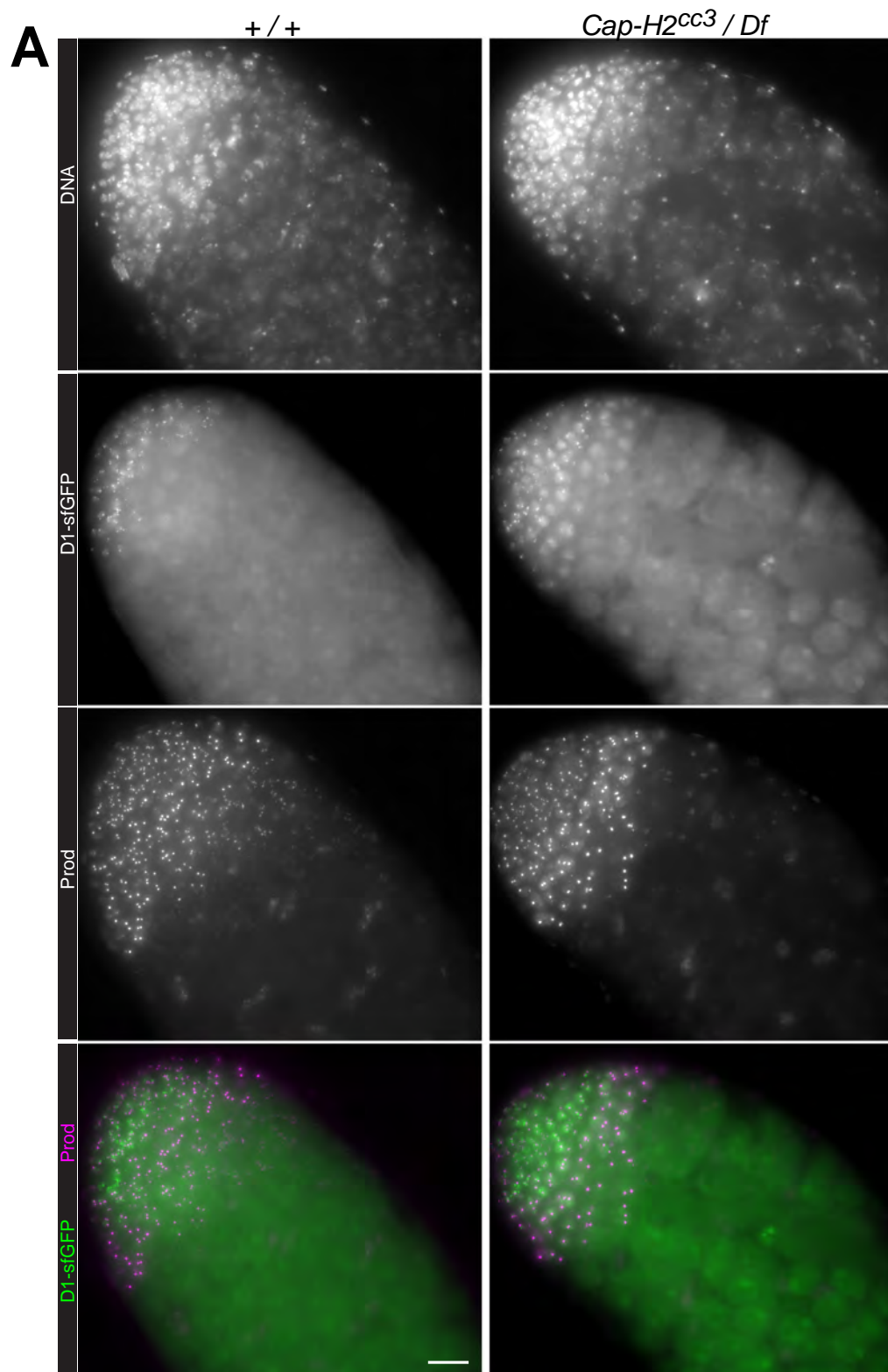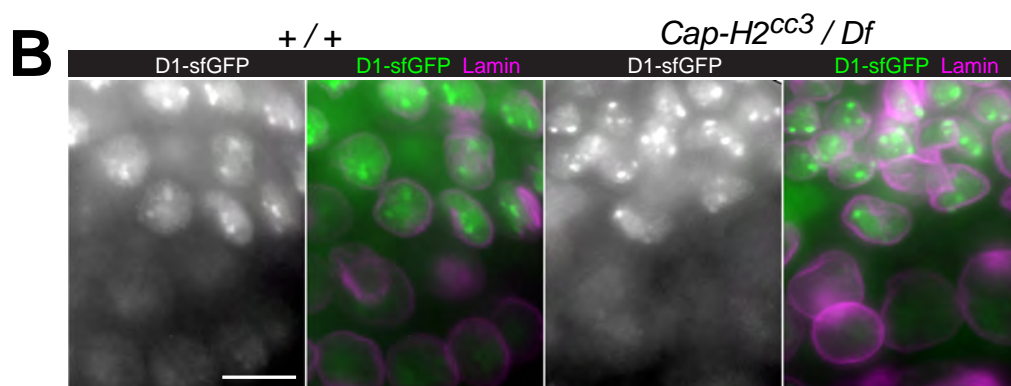

Supplement: S5 Fig — (A, B) Whole mount preparations of testes expressing D1-sfGFP were labeled with anti-Prod, anti-Lamin Dm0 and a DNA stain. Testes were either from control males (+ / +) of from Cap-H2cc3/ Df(3R)Exel6159 mutants. (A) Testis tip regions. (B) High magnification view with spermatogonial cells (upper region) and S1/2 spermatocytes (lower region) with high and low levels of D1-sfGFP, respectively. Scale bars = 20 μm (A) and 10 μm (B). (PDF) [file pgen.1009870.s005.pdf]

**A***bam* > *RedX*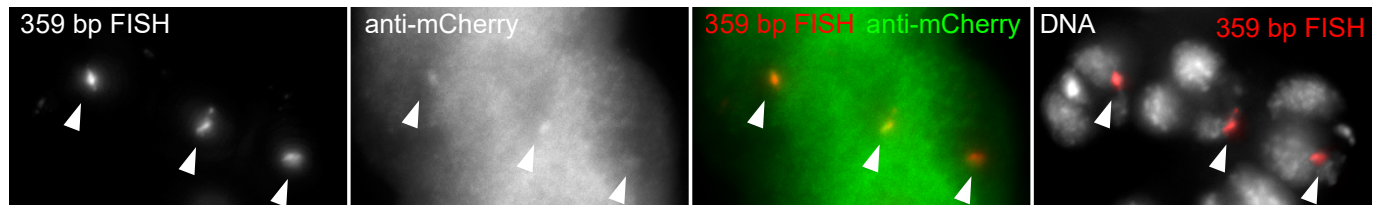*bam* > *Green2/3*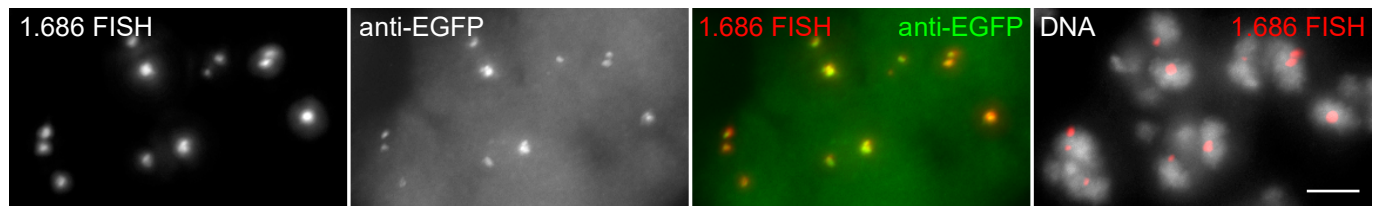**B***bam* > *RedX* *Green2/3*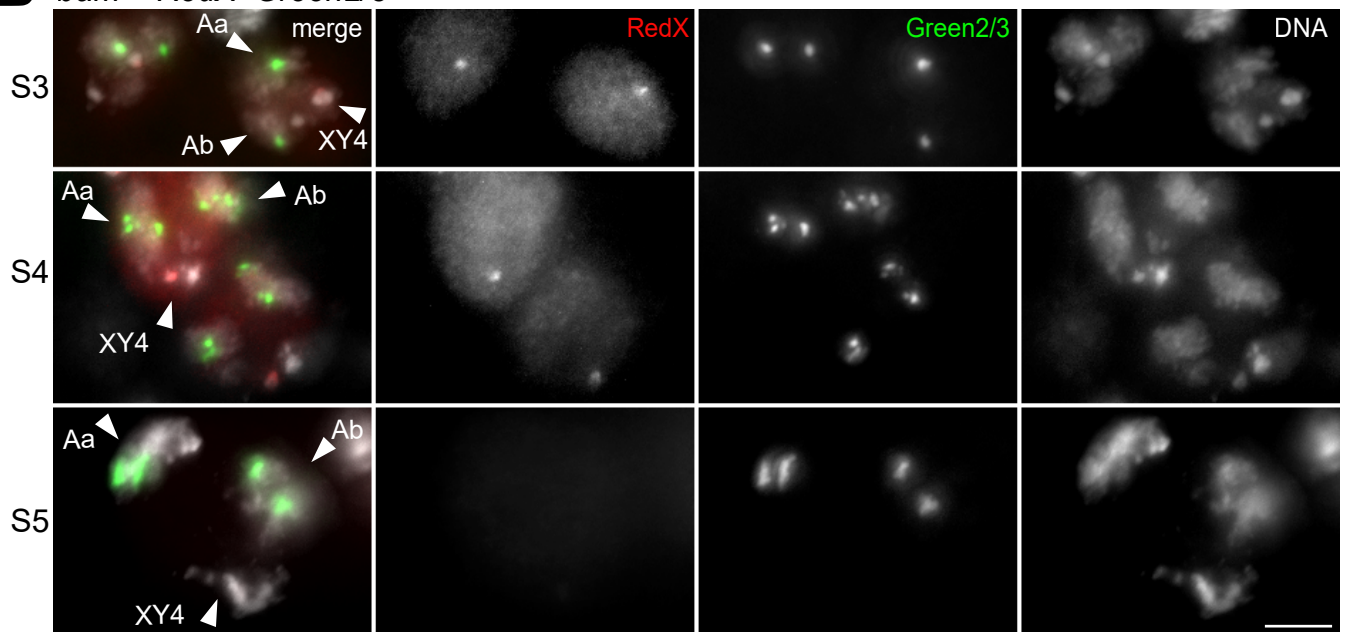

Supplement: S7 Fig — Squash preparations of testes from males with bamP-GAL4-VP16 and (A) either UASt-RedX (bam>RedX) or UASt-Green2/3 (bam>Green2/3), or (B) both together (bam>RedX Green2/3) were labeled with a DNA stain. (A) ImmunoFISH labeling. FISH probes targeting either the 359 bp satellite or the 1.686 satellite sequence were used in combination with either anti-mCherry for detection of RedX (arrowheads) or anti-EGFP for detection of Green2/3. High magnification views with S3 spermatocytes are displayed. (B) High magnification views with spermatocytes at the indicated stages are displayed. The three chromosome territories formed by the large autosomes chr2 and chr3 (Aa and Ab) and by the other chromosomes (XY4) are indicated on the left. Scale bars = 5 μm. (PDF) [file pgen.1009870.s007.pdf]
