# Supplementary material for: Bivalent individualization during chromosome territory formation in Drosophila spermatocytes by controlled condensin II protein activity and additional force generators
Source: PLoS Genet. 2021 Oct 20;17(10):e1009870. doi: 10.1371/journal.pgen.1009870 (PMC8559962; doi:10.1371/journal.pgen.1009870)
Supplement: S6 Fig — (A—C) Time-lapse imaging of His2Av-mRFP and Cenp-A/Cid-EGFP was applied for analysis of cyst cells in late spermatocyte cysts in (A) control (+ / +), (B) Cap-D3 mutants (Cap-D3EY/ Df), and (C) Cap-H2 mutants (Cap-H2cc3/ Df). Time (min:sec) is indicated relative to the onset of imaging. Scale bars = 3 μm. See S6 Fig for further explanations. (PDF) [file pgen.1009870.s006.pdf]

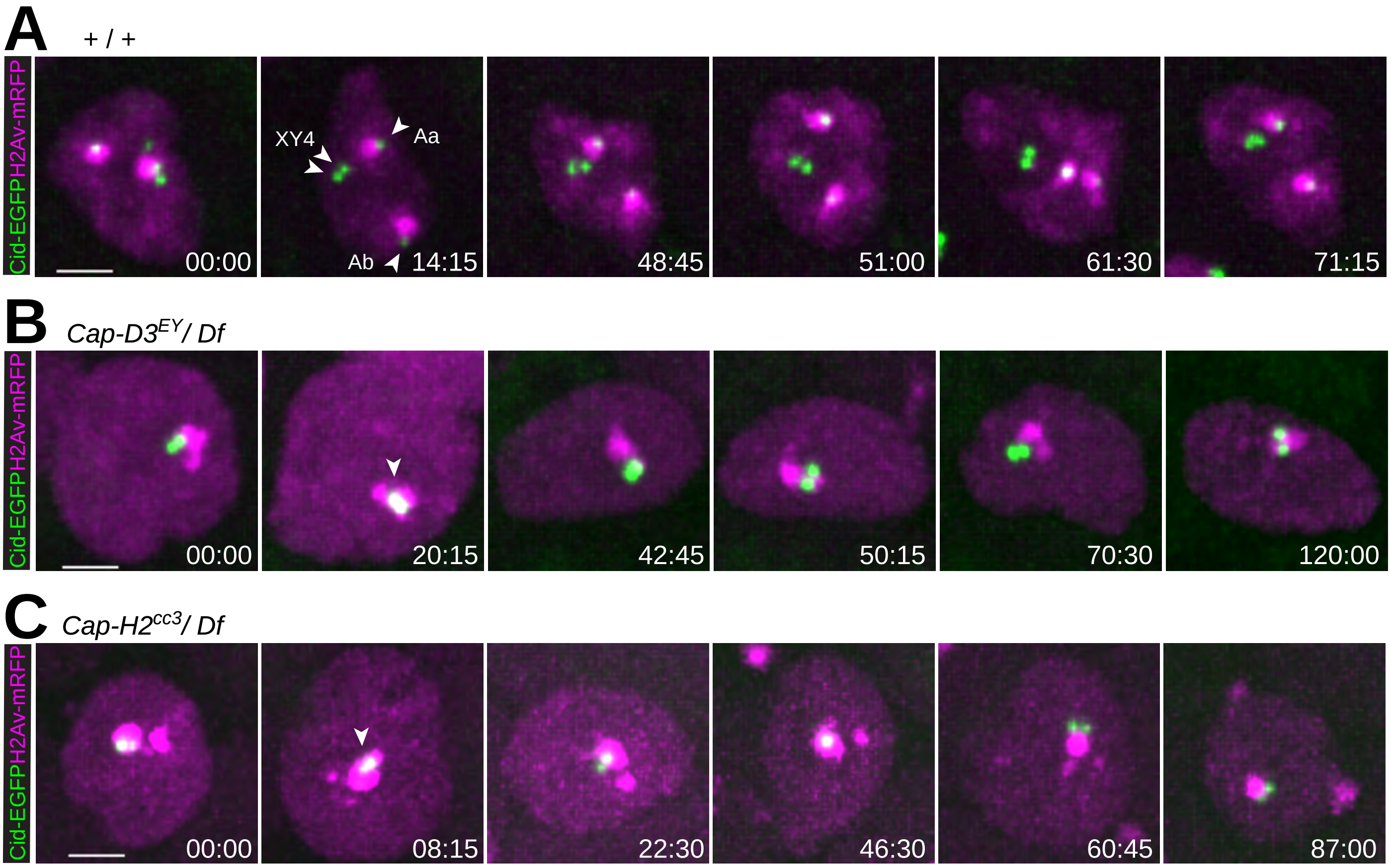

S6 Figure. Condensin II proteins are required for chromocenter and centromere cluster dissociation in somatic cyst cells.

(**A - C**) Time lapse imaging of His2Av-mRFP and Cenp-A/Cid-EGFP was applied for analysis of cyst cells in late spermatocyte. Time (min:sec) is indicated relative to the onset of imaging. Scale bars = 3 µm.

(**A**) In controls (+ / +), cyst cells displayed four Cid-EGFP dots in the nucleus during the S6 stage and during progression through M I. Two of these dots were in close proximity to each other, but well separated from the two additional dots. Each of these latter two dots was closely associated with a prominent His2Av-mRFP blob. Presumably, each of the four Cid-EGFP dots represents an unresolved pair of tightly associated homologous centromeres. The two Cid-EGFP dots close to His2Av-mRFP blobs (arrowheads Aa and Ab) are proposed to represent the clustered centromeres of chr2 and 3, respectively, adjacent to pericentromeric heterochromatin. Consequentially, the two closely paired Cid-EGFP dots (arrowheads XY4) are the paired chr4 centromeres and the paired centromeres of the sex chromosomes, respectively. A close spatial association of chr4 with sex chromosomes occurs also in spermatocytes and oocytes.

(**B,C**) in *Cap-D3* mutants (*Cap-D3*^{EY}/ *Df*) (**B**) and in *Cap-H2* mutants (*Cap-H2*^{cc3}/ *Df*) (**C**), only one or two closely adjacent Cid-EGFP dots were present (arrowheads). Moreover, these Cid-EGFP dots were brighter than in control cyst cells and next to only a single enlarged His2Av-mRFP blob. Thus, without condensin II proteins, cyst cell nuclei contain a chromocenter with an associated centromere cluster, while condensin II promotes the dissociation of chromocenter and centromere clusters in normal cyst cells.
