# Supplementary material for: Bivalent individualization during chromosome territory formation in Drosophila spermatocytes by controlled condensin II protein activity and additional force generators
Source: PLoS Genet. 2021 Oct 20;17(10):e1009870. doi: 10.1371/journal.pgen.1009870 (PMC8559962; doi:10.1371/journal.pgen.1009870)
Supplement: S8 Fig — See S8 Fig for further explanations. (PDF) [file pgen.1009870.s008.pdf]

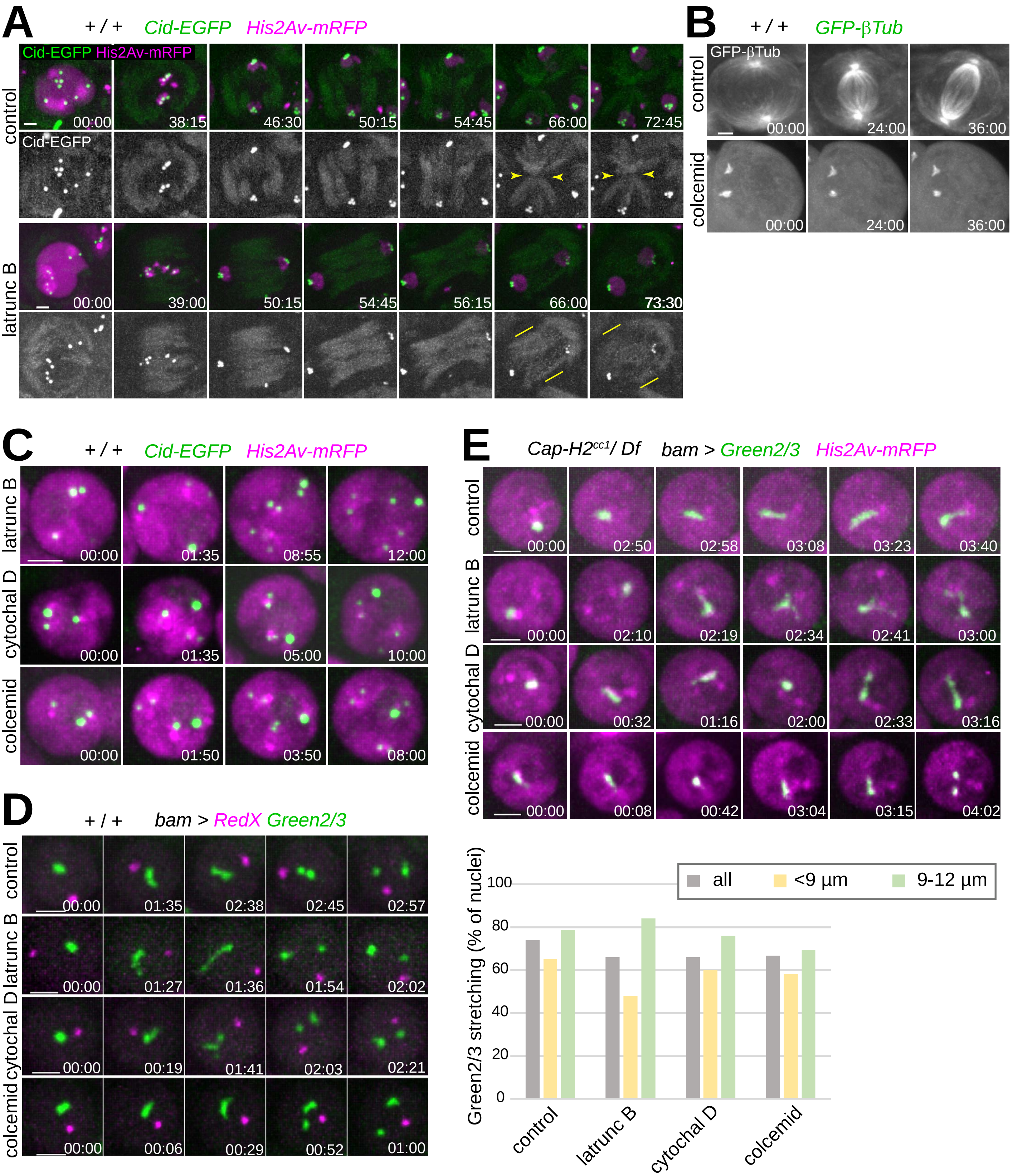

A
+ / + Cid-EGFP His2Av-mRFP
Cid-EGFP His2Av-mRFP
control
latrunc B
00:00
38:15
46:30
50:15
54:45
66:00
72:45
Cid-EGFP
39:00
56:15
73:30
B
+ / + GFP-βTub
GFP-βTub
colcemid
24:00
36:00
C
cytochal D
01:35
08:55
12:00
05:00
10:00
01:50
03:50
08:00
D
bam > RedX Green2/3
01:27
01:36
01:54
02:02
00:19
01:41
02:03
02:21
00:06
00:29
00:52
01:00
02:38
02:45
02:57
E
Cap-H2cc1/ Df bam > Green2/3 His2Av-mRFP
02:50
02:58
03:08
03:23
03:40
02:10
02:19
02:34
02:41
03:00
00:32
01:16
02:00
02:33
03:16
00:08
00:42
03:04
03:15
04:02
Green2/3 stretching (% of nuclei)
all
<9 µm
9-12 µm
0
20
40
60
80
100

S8 Fig. Inhibitors of F actin and microtubules do not interfere with chromosome territory formation in controls and with Green2/3 dot stretching in condensin II protein mutants

(**A, B**) Time lapse imaging of progression through M I confirms inhibitor effectivity. Spermatocytes expressing Cenp-A/Cid-EGFP and His2Av-mRFP were analyzed. Time (min:sec) is given relative to the onset of NEBD I. (**A**) The F actin inhibitor latrunculin B (latrunc B) results in a complete block of cytokinesis. Green autofluorescent signals primarily from mitochondria, which form a cylinder around the M I spindle, disclose contractile ring activity (yellow arrowheads) in mock treated (DMSO) control spermatocytes during exit from M I, but not in latrunculin B treated spermatocytes (yellow lines). Chromosome segregation, as indicated by the His2Av-mRFP and Cenp-A/Cid-EGFP signals is not affected by latrunculin B. A specific inhibition of cytokinesis was also observed in the presence of cytochalasin D. (**B**) Time lapse imaging of progression into M I in the presence of the microtubule inhibitor colcemid reveals a complete block of spindle formation. Untreated (control) and treated spermatocytes expressing GFP-βTub56D were analyzed.

(**C**) Inhibitors of F actin and MT dynamics do not preclude chromosome territory formation in *Drosophila* spermatocytes. Spermatocytes expressing His2Av-mRFP and Cenp-A/Cid-EGFP were treated with latrunculin B (latrunc B), cytochalasin D (cytochal D) or colcemid and analyzed by time-lapse imaging during the stage of chromosome territory formation. Time (h:min) is indicated.

(**D**) Inhibitors of F actin and MT dynamics do not preclude chromocenter disruption during chromosome territory formation. *bam>RedX Green2/3* spermatocytes were analyzed during the stage of chromosome territory formation in the absence (control, mock treated) or presence of the indicated inhibitors. Time-lapse imaging revealed an efficient splitting of single Green2/3 dots into two also in presence of the inhibitors. Time (h:min) is indicated.

(**E**) Stretching of 1.686 chromatin in condensin II protein mutants is not affected by inhibitors of F actin and MT dynamics. *Cap-H2* mutant spermatocytes (*Cap-H2*^{cc1}/*Df*) with *bam>Green2/3* and His2Av-mRFP were analyzed in the absence (control, mock-treated) or presence of the indicated inhibitors. Time lapse imaging revealed that Green2/3 dot stretching and definitive splitting into two occurs also in presence of inhibitors. Bar diagram indicating the percentage of *Cap-H2*^{cc1}/*Df* spermatocytes, which displayed at least one Green2/3 dot stretching or splitting episode during imaging for 4 hours in the indicated conditions. The analyzed spermatocytes were either at the earliest stages (d < 9 µM; S1 - S2) or thereafter (d = 9 -12 µM; S3 - S4). Latrunculin B resulted in an apparent slight reduction of Green2/3 stretching early but not late, whereas cytochalasin D and colcemid had at most very subtle effects. n = 126, 46, 80, 102, 52, 50, 70, 45, 25, 135, 31, 104 from left to right.
Time (h:min) is indicated.
Scale bars = 3 µm.
