# Supplementary material for: Bivalent individualization during chromosome territory formation in Drosophila spermatocytes by controlled condensin II protein activity and additional force generators
Source: PLoS Genet. 2021 Oct 20;17(10):e1009870. doi: 10.1371/journal.pgen.1009870 (PMC8559962; doi:10.1371/journal.pgen.1009870)
Supplement: S9 Fig — Scheme summarizing implicated mechanisms including explanations. (PDF) [file pgen.1009870.s009.pdf]

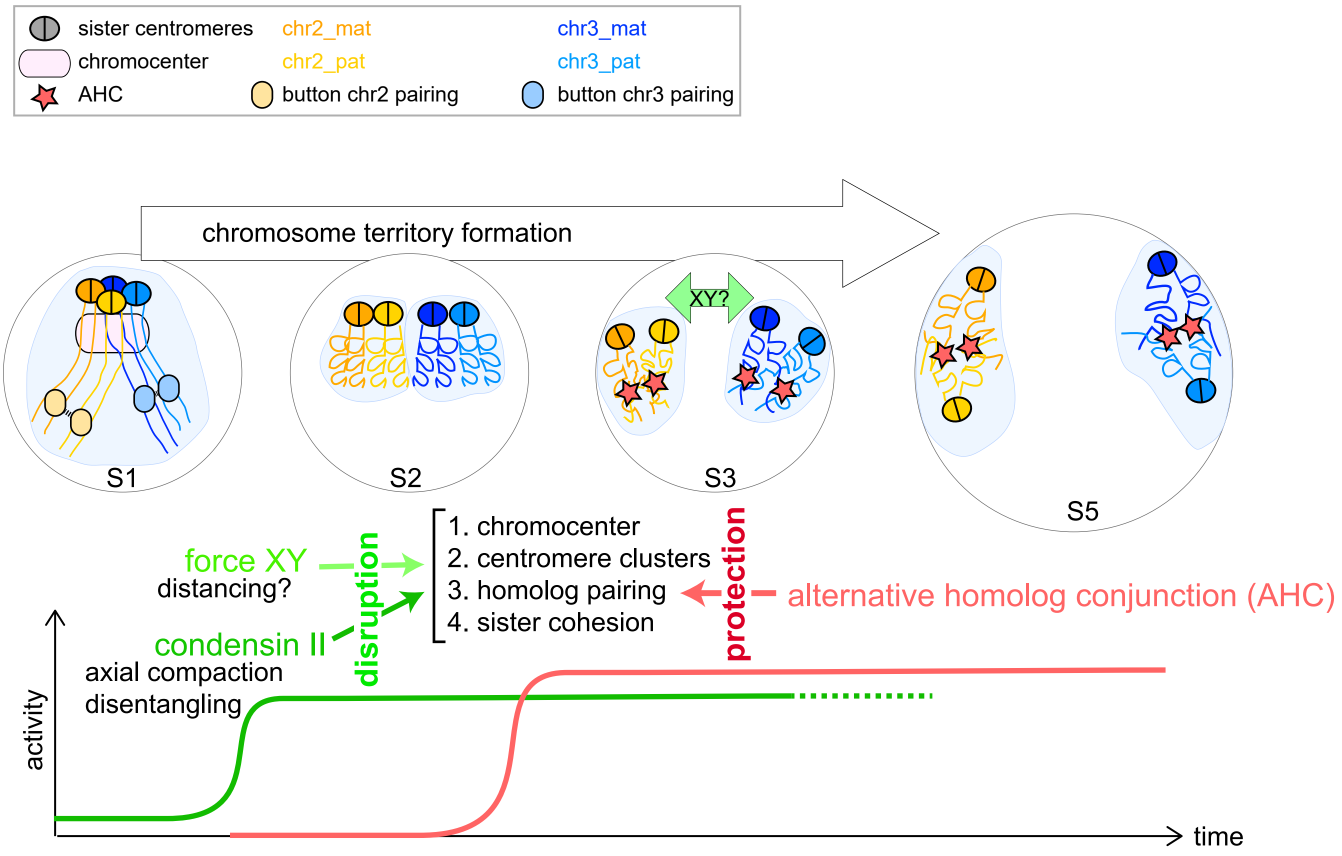

S9 Figure. Chromosome territory formation in *Drosophila* spermatocytes.

The scheme summarizes an updated speculative framework for the mechanisms that govern the process of chromosome territory formation in *Drosophila* spermatocytes. The scheme starts during S1 after completion of the meiotic S phase. For clarity, only the two large autosomes, chr2 and chr3, are considered, and only one chromosome arm of these metacentric chromosomes. Each arm is present in form of two sister chromatids (colored lines) after completion of the meiotic S phase. At the S1 stage, global chromosome organization in the interphase nucleus appears to be governed by a Rabl-like orientation that is established during anaphase of the last gonial mitotic division when all centromeres cluster close to the spindle pole. Protein-protein interactions of heterochromatin proteins in cis (along the chromatids) and in trans (between chromatids) compact the large, repeat-rich pericentric DNA sequence blocks of different chromosomes into a common chromocenter during early interphase. Moreover, homolog pairing in the euchromatic arm regions appears to be established rapidly, presumably by initial high-affinity interactions between homologous “button” regions occupied by locus-specific combinations of architectural and insulator proteins, followed by further zippering up. Only one of many such button regions per homolog is shown. As a result, during S1, chromosome intermingling is suggested to be relatively high within centromeric and pericentromeric regions and lower in euchromatic regions. During mid S2, a postulated increase in condensin II activity, possibly by increased expression of the limiting Cap-H2 subunit, initiates chromosome territory formation. Dynamic DNA loop extrusion by condensin II presumably rips apart trans interactions between chromatin proteins and results in axial chromosome compaction. Moreover, in combination with topoisomerase, DNA loop extrusion by condensin II might also promote DNA decatenation. Overall, the activities of condensin II and topoisomerase drive the individualization of each chromatid into a separate distinct territory. This chromatid disentangling is opposed by sister chromatid cohesion (cohesin) and the trans protein-protein interactions in centromeric, pericentromeric and euchromatic regions. Maximal disentangling activity in centromeric and pericentromeric regions might result in early disruption of chromocenter and centromere clusters

during S2b before wide-spread disruption of homolog pairing and sister chromatid cohesion in euchromatic regions during S3/4. Beyond condensin II, an unidentified force XY contributes to chromocenter disruption, and it might also explain the final wide spatial separation of territories with apparently chromatin-free gaps in between that cannot be generated conceivably by condensin II-driven DNA loop extrusion alone. After disruption of chromocenter and centromere clusters, alternative homolog conjunction (AHC) proteins are assembled in paired euchromatic regions in order to protect homolog pairing before complete disruption by condensin II and force XY. Homolog linkage by AHC appears to occur at a few spots along the chromosomes, but not at fixed invariable pairing sites, according to genetic and cytological analyses. Continued activity of condensin II and force XY during S3 and S4 increasingly separates centromeres and pericentromeric heterochromatin and disrupts also sister chromatid cohesion and homolog pairing except in regions protected by AHC. As a result, in a chromosome territory present in a mature S5 spermatocyte, homologous centromeres are widely separated apart with AHC protected regions of homolog pairing in between.

In the absence of condensin II activity, force XY is active but usually insufficient for definitive disruption of non-homologous associations within the chromocenter. In condensin II mutants, AHC is still assembled before and removed during M I. As AHC assembly presumably occurs primarily within euchromatic regions with minimal non-homologous intermingling, it makes a limited contribution to chromosome mis-segregation during M I in the condensin II mutants. In contrast, failure of disruption of chromocenter and centromere clusters causes massive M I mis-segregation in these mutants. Overexpression of Cap-H2 results in premature disruption of autosomal homolog pairing before assembly of AHC. As a result autosomes are present as univalents at the start of M I and thus segregate randomly.

We point out that the model above cannot be applied without amendments to the chromosomes omitted in this scheme (chr 4 and the sex chromosomes chrX and chrY). The small size of chr4 and the absence of extended euchromatic homology between chrX and chrY compensated by rDNA loci pairing likely explain the partially distinct behavior of these chromosomes during territory formation.
