# Supplementary material for: Bivalent individualization during chromosome territory formation in Drosophila spermatocytes by controlled condensin II protein activity and additional force generators
Source: PLoS Genet. 2021 Oct 20;17(10):e1009870. doi: 10.1371/journal.pgen.1009870 (PMC8559962; doi:10.1371/journal.pgen.1009870)
Supplement: Attachment — Submitted filename: ResponseToEditorReviewers.pdf [file pgen.1009870.s025.pdf]

**Response to editors**

1. "... we ask that you pay specific attention to the concerns that the paper could be significantly shortened."

While reviewer #3 considered our original version nearly flawless, reviewers #1 and #2 both suggested shortening. Therefore, we have revised the main text extensively to make it more concise, so that main findings, conclusions and take home messages are more accessible. Overall, the main text (introduction, results, discussion) was shortened by 15% and one of the main figures (Fig. 8 of the original version) was re-located into the supporting information (S8 Fig). As the changes in the text are so numerous, listing them in every detail would be rather impenetrable. However, we have uploaded a manuscript version with all changes tracked. In addition, the changes made in response to specific suggestions of the reviewers are explained below.

2. "The point regarding 4:4 segregation events that was raised by Reviewer 3" was addressed by inclusion of additional data into the text, as described in further detail below.

3. "All other comments of the reviewer" were addressed as described below.

**Response to reviewers**

<u>Reviewer #1</u>

We were pleased that this reviewer considered "data quality overall to be high" and our "discoveries of considerable interest". However, a clear recommendation was "to make the manuscript a bit more concise and to add clearer conclusions /take home messages more frequently throughout the manuscript", providing also a few suggestions. Although we appreciate and understand these, we note that in case of the specific example given for how figure titles should be written ("instead of 'time-lapse analysis of telomere behaviors', they should state 'telomeres do not cluster in Drosophila spermatocytes'") this particular recommendation was not considered, because Figure 2 documents that telomeres are clustered in early spermatocytes, at least partially, as well as that they are completely de-clustered at M I onset. In addition to the more global comments, this reviewer also made two specific comments. We fully agree to all comments and would like to thank reviewer #1 for these. As already stated above, we have revised the original text extensively to make it more concise. In addition, our response to the specific comments is as follows.

1. "Abstract is very reader-unfriendly. Chromocenter is not defined. Line 28-31 difficult to understand. 'persistence of centromere clusters ….. in the mutants…. do not depend on those proteins': which protein are they talking about?"

The abstract was re-written as suggested.

2. "Line 687-688: 'Figure 22A'?"

Fig. 6B was meant. The mistake was corrected.

<u>Reviewer #2</u>

This reviewer concluded that "this is an important paper" with strong "technical accomplishments". In addition, nine points were raised. We are grateful for these comments, which have given us very helpful advice for improving our manuscript.

1. "My most significant criticism is the length of this paper. The Introduction and Discussion are very long and should be shortened to make the paper more accessible. The first results section (and two

figures) are descriptive and only loosely connected to the rest of the paper. They do not involve the condensin mutants and the conclusions in line 397-398 are correlative and not based on direct testing of telomere function. The significance of Figure 2D and 2E is difficult to appreciate. In short, it seems the Results section could start with line 401 and figure 3."

As suggested, we have shortened introduction and discussion. However, after careful consideration of the suggested deletion of the initial part of the result section including Figs 1 and 2, we have decided to retain our original structure. The suggested deletion would undoubtedly increase the overall focus on condensin II, but this gain cannot make up for the associated loss of the results presented in Figs 1 and 2. These findings are among those that reviewer 3 has characterized as "other main results essentially negative but important nevertheless in clearing away incorrect or misleading ideas". For example, although Fig. 2 does not include "direct testing of telomere function" for chromosome territory formation, it presents novel and solid data on the dynamics of telomere localization in spermatocytes, indicating the telomere-driven rapid prophase movements comparable to those described in mammals and yeast do not occur during Drosophila male meiosis. Our results strongly argue against an important functional role of telomeres for homolog pairing and territory formation. While we have not deleted the initial parts, we have made them more concise.

2. "The dependence of the bridging phenotype on mnm is interesting. It might make sense to present the mnm data before UNO. That is, the mnm data shown that most of the bridges depend on AHC proteins. However, this might be indirect because UNO localization is not affected. In the same section, Fig 4 should include comparisons to the mnm single mutant. I also don't think the argument for "hypothetical ectopic AHC" is very strong. There is no evidence that the effect of mnm is due to ectopic AHC. Delete the discussion of Teflon (lines 625-631) because there is no data with it. Could move to discussion."

Evidently, the description of the *mnm* single mutant phenotype for comparison with condensin II mutants and with condensin II *mnm* double mutants was insufficient in the original version; reviewer #3 has made a similar comment. As explained in detail in our response to reviewer #3 (see below), we have made additional analyses with *mnm* single mutants and have incorporated new data into the text. Moreover, as suggested, the lines on *teflon* were largely deleted.

3. "Most observations and data well documented. But a few cases where the conclusions could be backed up with more quantitation. This includes lines 540-541 and line 821. Was EGFP-Cap-H2 overexpression verified with a measurement? (line 827)."

Quantification of the observations described in lines 540-541 is now included in the revised version (lines 500 – 503).

In case of the statement presented in line 821 of the original manuscript ("At the S3 stage, more than the normal number of three territories were displayed (Fig. 9B)"), we have not added a quantification. In contrast to this statement that refers to analyses with fixed testes, there is quantification of the observations made by time-lapse imaging (both in the original and the revised version). This quantification indicates that the absence of non-homologous 1.686 associations and of autosomal bivalents after EGFP-Cap-H2 overexpression is 100% penetrant. Therefore, we are convinced that inserting further quantification as suggested would impair readability without strengthening our conclusions substantially.

EGFP-Cap-H2 overexpression was not verified with a direct measurement. Unfortunately, we do not have the required tools. We have made neither an antibody against Cap-H2 nor an EGFP knock-in at the endogenous locus. Perhaps the antibody made by the Bosco lab (Hartl et al. 2008. Science) might be good enough for a direct demonstration of overexpression, but we note that in the publications of this group, this antibody was never used for direct verification of Cap-H2 overexpression. In this context, we point out that reviewer #3 has raised similar and additional questions concerning the EGFP-Cap-H2 overexpression experiments. Please see below for our responses.

4. “The section on stretching and splitting is confusing. It would benefit from a better definition of these two events, does it occur in wild-type, and its relationship or difference compared to territory formation. Stretching and splitting is introduced as something observed in the mutants (line 750) and Figure 7 lacks any measurement in wild-type. Where is the control data for Fig 7D-G. Is it in Fig 6C? Figure 8 has images of wild-type but the graph is for the mutant. Furthermore, my feeling is that the section from lines 765 – 806 adds little to the paper, especially if stretching and splitting is a mutant phenotype rather than a normal event. This paper has a few cases of negative results and this is probably the least informative of those.”

In early wild-type spermatocytes with a single Green2/3 dot, this dot is always split apart into two widely separated dots that will not re-associate again. Later the two dots are again split apart, resulting in spermatocytes with two pairs of dots. With time-lapse imaging, we have studied the first splitting event, the splitting of the single dots. In wild type, stretching of the single dot can be observed at the start of the splitting process, but only for a short time before separate daughter dots migrate far apart. In contrast, in condensin II mutants, splitting of the single dot into two daughter dots with a permanent wide separation as in wild type is rare (but not zero). Instead of the normal dot splitting process, stretching of the single dot over an extended time period can be observed. Stretching can result in periodic appearance of two bridged daughter dots followed by re-association. The temporal and spatial dynamics of the single dot stretching process in the mutants is variable. A simple classification of the single dot morphology as either stretched, partially split and completely split is impossible; a refined quantitative analysis with arbitrary but defined criteria would be extremely demanding. Fig. 6C indeed describes the wild-type and Fig 7D-G the mutant phenotypes. To us these findings are most simply explained by the suggestion that an unidentified force generator drives the separation of the non-homologous 1.686 satellite loci on chr2 and chr3, swiftly and successfully in wild type but not in condensin II mutants, where entanglements/associations cannot be resolved efficiently, resulting in prolonged stretching of the region with the non-homologous satellite associations. As suggested, the terms stretching and splitting and the phenotypes are more precisely described in the revised version.

Fig. 8 and lines 765 – 806 of our original version presented our results indicating that chromosome territory formation in wild-type, as well as the unidentified force generators responsible for the stretching of the region with the non-homologous 1.686 satellite associations in condensin II mutants do not depend on cytoskeletal dynamics (F actin, microtubules). Although our evidence is negative, we consider retention of the corresponding results in the manuscript far better than deletion. The literature on chromatin movements during interphase in meiotic and non-meiotic cells (cited in our original manuscript and in to a more limited extent in the discussion of the revised verion) makes a dependence of territory formation in wild-type and the chromatin stretching in condensin II mutant spermatocytes on cytoskeletal dynamics an obvious hypothesis that can be tested readily with specific drugs. In the original Fig. 8, it was panel A that showed the effects of drugs (latrunculin B, cytochalasin D, colcemid) in wild type and panel B in the *Cap-H2[cc1]* hemizygotes. These latter strong hypomorphs display a phenotype that can be enhanced and suppressed in principle, but our quantification does not reveal a phenotype modification in presence of the drugs. While our revised version does not delete this part, we have integrated the original Fig. 8 into the modified S9 Fig of the revised version in order to make the manuscript more concise. Note that “data not shown” is not permitted in PLoS Genetics.

5. “Lines 545-548 is confusing and should be deleted. Especially since the frequency of 4:4 segregation was so low. The fact that some of the 4:4 could also be defective could be more simply stated.”

Thanks. We have simplified the corresponding text (lines 507 – 509 in the revised version).

6. “Line 566: Is the additional reason for segregation errors a manifestation of the same defect in territory organization?”

Unfortunately, we do not understand this question. Hopefully, our revised text solves the issue.

7. “Line 344: “centromere s”

Thanks. corrected.

8. “Lines 569 – 586 is a distraction.”

We have shortened this part, moving most into supporting information (S6 Fig).

9. “Line 752: cc3/Df is written twice.”

Thanks. corrected.

Reviewer #3

Of course, we were very pleased with the extremely positive assessment of our original version by this reviewer who “found this manuscript to be outstanding in all respects”, “clearly written, indeed nearly flawless” with “innovative and well-designed experiments” addressing “interesting questions”, with results “clearly presented and fully support[ing] the conclusions”. We are also very grateful for all the minor criticism to which we have responded as follows:

1. “The significance of the authors’ statement that autosomal foci of UNO-EGFP could not be detected in CapD3 mutants (lines 608-610) is unclear. In a previous publication, this group showed that such foci could be unambiguously detected by over-exposure. In this study, did the authors try the over-exposure approach? The statement seems to suggest that they did but is not explicit.”

We have applied over-exposure, but it did not clarify whether autosomal UNO-EGFP dots are present or absent in *Cap-D3* mutants. The quite predictable central localization of the weak autosomal UNO-EGFP dots in particular in prometaphase I bivalents in wild-type supports their identification. This criterion cannot be used in the mutants, and this contributes to the difficulties in distinguishing potential specific signals from background in the mutants. We do not know whether autosomal UNO-EGFP dots are absent in *Cap-D3* mutants or present but not detectable for technical reasons. The revised version includes a more detailed presentation of this issue (lines 553 – 557).

2. “The question of the presence or absence of autosomal AHC proteins is of even more interest in the Cap-H2 overexpression genotype where autosomal conjunction is absent. Does overexpression of Cap-H2 lead to absence of AHC proteins on autosomes? Or does autosomal pairing fail despite presence of AHC proteins? It would seem this question could be answered by testing for UNO-EGFP foci by over-exposure.”

We fully agree that the question of whether autosomal AHC protein dots are present or absent after Cap-H2 overexpression is of considerable interest. So far, we have not addressed it. Reliable detection of the weak autosomal dots in wild type depends on expression of EGFP-tagged AHC proteins. Signals obtained with mCherry tagged proteins are not sufficiently strong. Based on our experience with the technical difficulties of observing the weak autosomal AHC protein dots, the expression of EGFP-tagged AHC proteins in combination with overexpression of EGFP-Cap-H2 is unlikely to be successful because of an inability to differentiate EGFP-tagged AHC proteins from EGFP-Cap-H2. We do not have UASt transgenes for expression of untagged Cap-H2. The overexpression resulting with Cap-H2[EY09979] (an enhancer trap insertion introducing UASp upstream), which has been used for the publication from the Bosco lab, results in a far milder phenotype. Generating promising tools for analysis of this issue would require many months of additional work.

3. “What is the significance of the disappearance of CapH2-EGFP around stage S3? Is this an abnormal property of the tagged protein or does it reflect the dynamics of the wild-type protein? It would be useful to know if CapH2-EGFP rescues CapH2 loss-of-function mutants. If CapH2-EGFP is a normal, functional CapH2, one would expect absence of both the loss-of-function and over-expression phenotypes in the rescue genotype. This would increase confidence that the reported results with respect both to protein stability and OE phenotypes are not artifacts of an abnormal protein.”

We agree that additional information concerning the functional equivalence of EGFP-Cap-H2 and Cap-H2 would be helpful. The effects of *bam-GAL4-VP16* driven expression of EGFP-Cap-H2 in spermatocytes are rather similar to those observed after expression of untagged Cap-H2 from Cap-H2[EY09979] with other GAL4 drivers (Hsp70-GAL4) in other tissues (nurse cells in ovaries, larval salivary glands) that were reported in the publications of the Bosco lab (Hartl et al. 2008. Science; Bauer et al. 2012). Axial compaction of chromosomes and disruption of centromere clusters during interphase results with both tagged and untagged Cap-H2, suggesting that the overexpression phenotypes do not simply result from an interference of EGFP with normal Cap-H2 function. Accordingly, the *bam>EGFP-Cap-H2* phenotype is caused presumably by higher levels of a protein that has normal function. A convincing analysis of EGFP-Cap-H2 functionality would require time-consuming generation of additional tools (like EGFP knock-in at the endogenous locus and generation and characterization of high quality anti-Cap-H2). Mutant rescue experiments with our *UASt-EGFP-Cap-H2* transgenes cannot give definitive answers. In all likelihood, *UASt-EGFP-Cap-H2* expression achieved with various *GAL4* drivers will not correspond to that of the endogenous *Cap-H2* gene with regard to temporal profile, tissue specificity and resulting protein levels. In the revised version, we have clearly stated limitations of our current evidence concerning functionality of EGFP-Cap-H2 in the discussion (lines 842 – 846).

4. “An important control is missing in Fig. 4H: the frequency of 4:4 segregations in mnm single mutants. Without it, it is not obvious whether the low frequency in the double mutants means anything. Readers may wonder if the loss the conjunction and resulting random assortment alone would suffice to explain it. In fact it does not – assuming random assortment (which is pretty close to reality for mnm null mutants), the probability that 8 univalents will segregate 4:4 is .27 (from the binomial expansion), much higher than the reported double mutant frequency, which appears from the graph to be around 5%. At minimum, it would be helpful to point out how different the observed results are from the theoretical expectation. This strongly suggests that the defects in bivalent orientation caused by condensin II mutants do not affect segregation solely by causing random assortment. The outcome is even worse than random assortment! It will be interesting to learn more about it.”

We have made additional analyses of anaphase I in *mnm* single mutants and have incorporated new data on the frequency of 4:4 segregation in these single mutants into the text (lines 591 - 594). Our results on the pattern of centromere segregation in *mnm* single mutants during M I (see below) are in excellent agreement with random assortment, as expected. 4:4 segregation was observed in 25%, close to the expected 27%.

| segregation pattern | prediction in case of random segregation of 8 kts | | observed in $mnm^{z3\text{-}3298}/mnm^{z3\text{-}5578}$ | |
|---|---|---|---|---|
| | binomial co-efficients | % | number of daughter cell pairs | % |
| 4:4 | 70 | 27.3 | 11 | 25.0 |
| 5:3 | 112 | 43.8 | 19 | 43.2 |
| 6:2 | 56 | 21.9 | 11 | 25.0 |
| 7:1 | 16 | 6.3 | 3 | 6.8 |

| | | | | |
|---|---|---|---|---|
| 8:0 | 2 | 0.8 | 0 | 0.0 |
| sum | 256 | 100 | 44 | 100 |

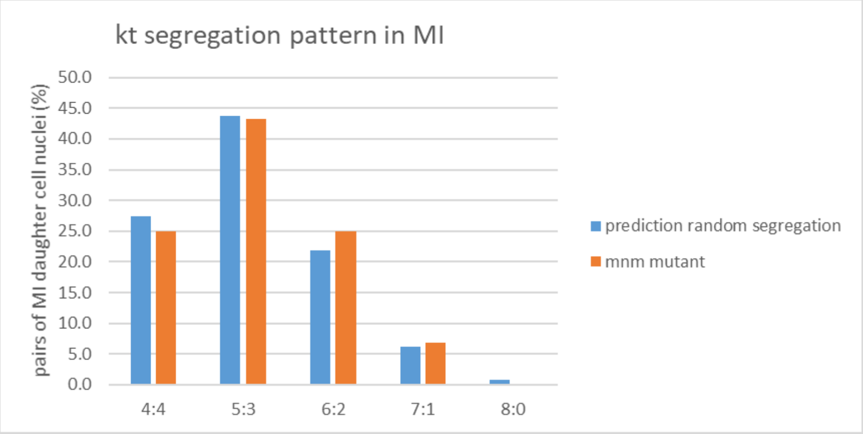


5. “This is very minor but it was not clear to me that condensin II has much effect on sister chromatid cohesion, at least in this study. So I was not sure what the authors had in mind by including cohesion in their summary figure.”

Unfortunately, we have submitted an incomplete version of the summary figure (S9 Fig.) along with the original manuscript. This incomplete S9 Fig did not contain an explanatory text. The revised S9 Fig, which was modified slightly, now includes this explanatory text.

The inclusion of cohesion is based on the results of Vazquez et al. (2002) and Tsai et al. (2011), showing that in addition to the striking and extensive loss of homolog pairing during spermatocyte maturation there is also substantial loss in sister chromatid cohesion. We speculate that sister separation might also be driven by the activity of condensin II perhaps in combination with force xy.

6. Editorial corrections

- Line 469: use numerical citation

Thanks. corrected

- Lines 687-688: Figure 22A should presumably be 6A.

Thanks. corrected

- Line 798: should be “Similar results were obtained with …..”

Thanks. corrected

- Lines 847-848: Authors presumably mean “Not only lack of…” or “Not only loss of….”

Thanks. corrected
