# Supplementary material for: Bivalent individualization during chromosome territory formation in Drosophila spermatocytes by controlled condensin II protein activity and additional force generators
Source: PLoS Genet. 2021 Oct 20;17(10):e1009870. doi: 10.1371/journal.pgen.1009870 (PMC8559962; doi:10.1371/journal.pgen.1009870)
Supplement: Attachment — Submitted filename: Review of Vernizzi & Lehner_mckee.docx [file pgen.1009870.s024.docx]

Comments on Vernizzi & Lehner

I found this manuscript to be outstanding in all respects. The experiments address interesting questions and are innovative and well-designed. The results are clearly presented and fully support the conclusions. The manuscript itself is clearly written, indeed nearly flawless. I especially enjoyed the very informative introduction and the beautiful figures and movies. I think it is important that this study focused on male meiosis, where condensin II activity is essential. There have been quite a few excellent studies of condensin II function in Drosophila over the past two decades but nearly all have used either cultured cells or focused on other cell types where condensin II is dispensable.

Drosophila male meiosis is unique in several ways and thus a bit out of the mainstream, so a few paragraphs summarizing and emphasizing the importance of the main findings might be helpful.

The most important finding is that condensin II activity is essential to eliminate centromere clustering and resolve chromocenters prior to the onset of the meiosis I division, and that failure to achieve chromocenter resolution in cap-H2 and cap-D3 mutants disrupts bivalent bi-orientation and causes very high frequencies of homolog mis-segregation. The mis-segregation frequency is unchanged by simultaneous mutation of mnm, so is independent of conjunction and independent of the frequency of chromosome bridges (80% of which are eliminated by the mnm mutations). Previous studies of condensin II in spermatocytes focused on these bridges and led to the view that mis-segregation is largely due to unresolved chromatin interlocks. The authors’ results suggest instead that mis-segregation in condensin II mutants is probably not driven largely by ectopic chromatin interlocks but rather by chromocenter persistence. To my knowledge, this observation of a disruptive role of chromocenters on bivalent orientation is novel and may apply to other systems besides Drosophila meiosis, as chromocenters are universal features of cells.

Another important finding is that the anti-pairing effect of condensin II previously observed in other Drosophila cell types applies also to spermatocytes, where the authors showed that Cap-H2 overexpression disrupts pairing of autosomal homologs from the onset of meiosis and through the meiosis I division (even though Cap-H2-EGFP is largely removed by mid-prophase). The finding, based on the TALE light system, of disrupted pairing in S1/S2 does need to be interpreted with some caution because it is based solely on analysis of a single heterochromatic locus; it will be important to learn whether general euchromatic pairing during S1/S2 is similarly disrupted. If confirmed, however, an important implication will be that condensin II anti-pairing does not affect only conjunction-mediated pairing, because conjunction is not required (and may not exist) prior to S3. It can also effectively oppose the currently unknown forces that mediate meiotic pairing in early prophase. Homolog pairing remains poorly understood in general so this insight could contribute to its elucidation.

Several of the other main results in the study are essentially negative but important nevertheless in clearing away incorrect or misleading ideas: the refutation of the idea that there is a defined stage of obligate centromere pairing after the dissolution of chromocenters in stage S3; the evidence against a major role for cytoskeletal proteins in spermatocyte territory formation; and the failure to find support for major roles of the D1 and Prod proteins in territory formation in spermatocytes.

A potentially important technical innovation is the TALE light system, which essentially substitutes for FISH in live imaging studies, where FISH cannot be carried out. The results for the two tested loci were a bit mixed, since the 359 signal petered out in mature spermatocytes, but the 1.686 probe worked very well and both probes made possible a number of interesting findings.

My only criticisms are relatively minor, but addressing them would strengthen the manuscript.

Minor criticisms

The significance of the authors’ statement that autosomal foci of UNO-EGFP could not be detected in CapD3 mutants (lines 608-610) is unclear. In a previous publication, this group showed that such foci could be unambiguously detected by over-exposure. In this study, did the authors try the over-exposure approach? The statement seems to suggest that they did but is not explicit.

The question of the presence or absence of autosomal AHC proteins is of even more interest in the Cap-H2 overexpression genotype where autosomal conjunction is absent. Does overexpression of Cap-H2 lead to absence of AHC proteins on autosomes? Or does autosomal pairing fail despite presence of AHC proteins? It would seem this question could be answered by testing for UNO-EGFP foci by over-exposure.

What is the significance of the disappearance of CapH2-EGFP around stage S3? Is this an abnormal property of the tagged protein or does it reflect the dynamics of the wild-type protein? It would be useful to know if CapH2-EGFP rescues CapH2 loss-of-function mutants. If CapH2-EGFP is a normal, functional CapH2, one would expect absence of both the loss-of-function and over-expression phenotypes in the rescue genotype. This would increase confidence that the reported results with respect both to protein stability and OE phenotypes are not artifacts of an abnormal protein.

An important control is missing in Fig. 4H: the frequency of 4:4 segregations in mnm single mutants. Without it, it is not obvious whether the low frequency in the double mutants means anything. Readers may wonder if the loss the conjunction and resulting random assortment alone would suffice to explain it. In fact it does not – assuming random assortment (which is pretty close to reality for mnm null mutants), the probability that 8 univalents will segregate 4:4 is .27 (from the binomial expansion), much higher than the reported double mutant frequency, which appears from the graph to be around 5%. At minimum, it would be helpful to point out how different the observed results are from the theoretical expectation. This strongly suggests that the defects in bivalent orientation caused by condensin II mutants do not affect segregation solely by causing random assortment. The outcome is even worse than random assortment! It will be interesting to learn more about it.

This is very minor but it was not clear to me that condensin II has much effect on sister chromatid cohesion, at least in this study. So I was not sure what the authors had in mind by including cohesion in their summary figure.

Editorial corrections

Line 469: use numerical citation

Lines 687-688: Figure 22A should presumably be 6A.

Line 798: should be “Similar results were obtained with …..”

Lines 847-848: Authors presumably mean “Not only lack of…” or “Not only loss of….”
